# Interactions of the human cardiopulmonary, hormonal and body fluid systems in parabolic flight

**DOI:** 10.1007/s00421-014-2856-3

**Published:** 2014-03-13

**Authors:** U. Limper, P. Gauger, P. Beck, F. Krainski, F. May, L. E. J. Beck

**Affiliations:** 1 Department of Anesthesiology and Surgical Intensive Care Medicine, Merheim Medical Center, Hospitals of Cologne, University Witten/Herdecke, Ostmerheimer Strasse 200, 51109 Cologne, Germany; 2 Department of Space Physiology, Institute of Aerospace Medicine, German Aerospace Center (DLR), Cologne, Germany; 3 Department of General Surgery, Helios Hospital Wuppertal, University Witten/Herdecke, Wuppertal, Germany; 4 University of Texas Southwestern Medical School, Internal Medicine, Dallas, TX USA

**Keywords:** Inert gas rebreathing, Weightlessness, Hypobaric hypoxia, Hypobaric chamber, Gravity

## Abstract

**Purpose:**

Commercial parabolic flights accessible to customers with a wide range of health states will become more prevalent in the near future because of a growing private space flight sector. However, parabolic flights present the passengers’ cardiovascular system with a combination of stressors, including a moderately hypobaric hypoxic ambient environment (HH) and repeated gravity transitions (GT). Thus, the aim of this study was to identify unique and combined effects of HH and GT on the human cardiovascular, pulmonary and fluid regulation systems.

**Methods:**

Cardiac index was determined by inert gas rebreathing (CI_rb_), and continuous non-invasive finger blood pressure (FBP) was repeatedly measured in 18 healthy subjects in the standing position while they were in parabolic flight at 0 and 1.8 G_z_. Plasma volume (PV) and fluid regulating blood hormones were determined five times over the flight day. Eleven out of the 18 subjects were subjected to an identical test protocol in a hypobaric chamber in ambient conditions comparable to parabolic flight.

**Results:**

CI_rb_ in 0 G_z_ decreased significantly during flight (early, 5.139 ± 1.326 L/min; late, 4.150 ± 1.082 L/min) because of a significant decrease in heart rate (HR) (early, 92 ± 15 min^−1^; late, 78 ± 12 min^−1^), even though the stroke volume (SV) remained the same. HH produced a small decrease in the PV, both in the hypobaric chamber and in parabolic flight, indicating a dominating HH effect without a significant effect of GT on PV (−52 ± 34 and −115 ± 32 ml, respectively). Pulmonary tissue volume decreased in the HH conditions because of hypoxic pulmonary vasoconstriction (0.694 ± 0.185 and 0.560 ± 0.207 ml) but increased at 0 and 1.8 G_z_ in parabolic flight (0.593 ± 0.181 and 0.885 ± 0.458 ml, respectively), indicating that cardiac output and arterial blood pressure rather than HH are the main factors affecting pulmonary vascular regulation in parabolic flight.

**Conclusion:**

HH and GT each lead to specific responses of the cardiovascular system in parabolic flight. Whereas HH seems to be mainly responsible for the PV decrease in flight, GT overrides the hypoxic pulmonary vasoconstriction induced by HH. This finding indicates the need for careful and individual medical examination and, if necessary, health status improvement for each individual considering a parabolic flight, given the effects of the combination of HH and GT in flight.

**Electronic supplementary material:**

The online version of this article (doi:10.1007/s00421-014-2856-3) contains supplementary material, which is available to authorized users.

## Introduction

Parabolic flights performed in slightly modified passenger airplanes operating in the troposphere have been used extensively in past decades for space-related human physiological research. Most of these life science experiments have been conducted on parabolic flights in a KC 135 aircraft in the USA or in an Airbus A300 aircraft in Europe. Both aircrafts achieve parabolic trajectories of different sequences. The A300 performs 31 parabolas each flight day, with a 2-min break of level flight between two consecutive parabolas and with 4-min breaks after each group of five parabolas. After the 16th parabola, a break of 8 min represents half-time of the flight. The KC 135 performed 40 parabolas per flight day and took a so-called roller coaster flight path with 10 parabolas back-to-back. Typically a KC 135 flight had 5-min breaks after the 10th and the 30th parabola and a 10-min break after the 20th parabola. Extensive previous research was aimed at investigating the cardiovascular system in the context of changing gravity in general and weightlessness in particular (Liu et al. 2012; Mukai et al. 1991; Petersen et al. 2011). Most of these experiments focused on cardiovascular responses during gravity transitions (Limper et al. 2011; Mukai et al. 1994). Fewer studies have investigated longitudinal changes in these specific cardiovascular responses over the course of a parabolic flight day. Only Mukai et al. reported longitudinal changes in cardiac index (CI), although concrete CI differences were not reported. In particular, Mukai et al. used impedance cardiography during the first 10 parabolas of a parabolic flight. Mukai et al. (1994) also demonstrated a decreased thoracic fluid index by thoracic impedance measurements, and they noted that such a decreased thoracic fluid index may indicate an increase in thoracic fluids during parabolic flight. However, to date, no research has investigated how the body fluid system is influenced by parabolic flights, although some evidence suggests that the intravascular volume may increase on the day of a parabolic flight, as stated by Schlegel et al. (2001).

Another important factor that has not been adequately addressed in prior experiments is the changing ambient atmosphere of the airplane cabin on the day of a flight. This seems astonishing because of the tremendous amount of work which has been carried out on the effects of hypoxia of commercial air travel on the human body (Gradwell 2006; Mortazavi et al. 2003). An inflight cabin atmosphere that is more hypobaric, hypoxic and dry with respect to ground control may affect the responses of the cardiovascular system, particularly to changes in gravity. The ambient pressure of the European A300 at cruising altitude is approximately 830 mbar, which is equal to the ambient pressure at 1,650 m above sea level (m. a. s. l.) (Lehot 2012). The ambient pressure of the KC 135, which is no longer in service, was 751 mbar, which is equal to an altitude of 2,438 m. a. s. l. (Lehot 2012).

Even the mild hypobaric hypoxia (HH), equivalent to 2,400 m. a. s. l., of a typical airplane cabin has been associated with a reduction of baroreflex sensitivity (Sevre et al. 2002), which is one of the most important cardiovascular control mechanisms under changing gravity. In contrast, hypoxemia with an arterial oxygen saturation of 90–95 % causes pulmonary vasoconstriction, leading to a 20 % increase in pulmonary pressure in healthy subjects during air travel (Smith et al. 2012). Hypoxic pulmonary vasoconstriction (HPV) leads to a reduction of the arterial and venous blood volume in the lungs (Sylvester et al. 2012). Consequently, we can expect opposing mechanisms to act on the pulmonary blood volume during parabolic flight: a cephalic volume shift under microgravity bouts would increase pulmonary blood volume, whereas the persistent HPV would decrease it. Furthermore, it is known today that even slight hypobaric hypoxic conditions at quite low altitudes, from 1,000 m. a. s. l., induce changes in blood volume (Bartsch and Saltin 2008). However, the effects of HH on baroreflex sensitivity, pulmonary blood volume and the body fluid system during parabolic flights have not yet been examined.

We therefore measured cardiovascular, pulmonary, hormonal and fluid volume parameters during parabolic flights and repeated these measurements in a hypobaric chamber. Our specific hypotheses were the following: (1) cardiac output in a state of weightlessness is not constant during a parabolic flight but rather increases over time because of an increase in intravascular volume; and (2) the cephalic blood volume shift in weightlessness overrules the hypoxic pulmonary vasoconstriction and leads to an increase in lung tissue volume.

When designing this study, we also considered the commercial airplane parabolic flights and upcoming suborbital commercial parabolic flights. We believe that more research must be done to clarify potential health issues that may arise from the combined effect of moderate hypobaric hypoxia and intense gravitational transitions, particularly for flight surgeons responsible for future, most likely elderly, customers of airplane and suborbital parabolic flights.

## Methods

### Subjects

Eighteen healthy subjects participated in the parabolic flight study, and 11 also repeated an identical test protocol in the hypobaric chamber of the German Aerospace Center (DLR) in Cologne, Germany, during the first 3 months after their flights (Table 1). The test protocols were approved by the pertinent authorities (a) for the parabolic flights: *agence française de sécurité sanitaire des produits de santé* and *comité de protection des personnes nord oeust III* and (b) for the hypobaric chamber experiments: *Ärztekammer Nordrhein*. All subjects were free of any cardiopulmonary, renal or other systemic diseases, none were taking any medications on a regular basis and each passed a special parabolic flight medical examination (requirements of the parabolic flight executing company, (NOVESPACE 2013)) based on the JAR Class III examination at the aeromedical center of the DLR, Cologne. All subjects provided written informed consent to participate in the study. Heavy exercise and alcohol were strictly prohibited beginning 24 h before any testing. Scopolamine-hydrobromide was applied subcutaneously before the flights (125 μg in women and 175 μg in men) as a prophylactic against motion sickness. The same dosage was also administered before the hypobaric chamber tests to allow for comparable test conditions (Hyoscine Injection BP 400 µg/ml, UCB Pharma Ltd, Berkshire, UK). Subjects drank between 100 and 200 ml of water during an experiment day to antagonize dry mouth caused by scopolamine medication and rebreathing maneuvers.

**Table 1:** Subject characteristics

No.	Subject code	Sex	Height (cm)	Weight (kg)	BMI (kg/m^2^)	Age (year)	First flyer	M.S.	Hypobaric chamber
1	0AA	M	179	80	25	28	N	N	Y
2	0AC	F	172	58	20	44	N	N	Y
3	0AL	M	181	72	22	28	N	N	Y
4	0AM	F	172	67	23	27	N	N	Y
5	0AO	F	158	50	20	33	Y	N	Y
6	0AK	M	168	70	25	45	N	N	Y
7	0AD	M	182	75	23	28	N	Y	Y
8	0AP	M	191	80	22	44	Y	Y	Y
9	0AR	M	172	67	23	43	N	N	N
10	0AS	F	167	58	21	31	Y	N	Y
11	0AW	F	167	55	20	29	Y	N	Y
12	0AX	F	167	60	22	29	N	N	Y
13	0AT	F	163	52	20	34	Y	Y	N
14	0BF	M	165	58	21	36	Y	N	N
15	0BR	M	179	81	25	36	Y	N	N
16	0BX	F	172	66	22	30	Y	N	N
17	0BY	F	167	59	21	28	Y	N	N
18	0CC	M	175	80	26	30	N	N	N
Ratio		9:9					9:9	15:3	18:11
Mean			172	66	22	34			
SD			8	10	2	6			

*M* male, *F* female, *N* no, *Y* yes, *BMI* body mass index, *First flyer* subject had never participated in a parabolic flight before, *M.S.* motion sickness experienced by the subject, *Hypobaric chamber* subject participated in the supplemental hypobaric chamber experiment

### Experiment protocols

#### Parabolic flights

Data were obtained during the 15th, 16th and 19th DLR parabolic flight campaigns between 2010 and 2012. The flights were performed in the Airbus A300 Zero-G of the French NOVESPACE company in Bordeaux, France. Flights took off from and returned to the Bordeaux Merignac Airport (airport altitude: 49 m. a. s. l.) where the pre- and post-flight measurements were performed. Each flight campaign consisted of three successive flight days. Thirty-one parabolas were flown on each flight day in sets of five consecutive parabolas separated by short 4–5 min phases of steady flight. The 16th and 17th parabolas were separated by a longer, 8-min break. During the flights, the cabin environmental conditions were as follows: 830 mbar pressure (equivalent to an altitude of 1,650 m. a. s. l.), approximately 15 % humidity, an ambient temperature of approximately 19 °C, an illumination level of approximately 800 lux, a light color temperature between 3,400 and 3,600 K, a noise level of 70–80 dB and a vibration level of approximately 0.008 g with a frequency spectrum of 1–400 Hz. The ambient atmospheric conditions on the ground during pre- and post-flight varied over the time period of the campaigns because of changes in weather conditions and seasons; the ambient pressure was approximately 1,005 mbar, the humidity ranged from 30 to 100 % and temperature ranged from 9 to 26 °C.

**Fig. 1:**
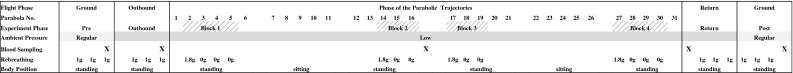
Study design: measurements were performed at regular ambient pressure before and after parabolic flight and hypobaric chamber runs (pre and post, respectively); and under low ambient pressure conditions in a standing position in a parabolic flight and in the hypobaric chamber (outbound, block 1–4, return); and in a standing position combined with gravity transitions in parabolic flight and without gravity transitions in the hypobaric chamber. Measurement blocks for cardiovascular and pulmonary data acquisition and time points of blood sampling are indicated

After a light breakfast, each subject was equipped with a lead-II electrocardiogram (ECG), impedance cardiogram (ICG) and finger blood pressure device (FBP). Thereafter, an indwelling short 16 G catheter for blood sampling was inserted in the antecubital vein of the right arm (Vasofix^®^ Certo, B. Braun Melsungen AG, Melsungen, Germany). Subsequently, subjects received scopolamine at 8 a.m., and a baseline blood sampling was performed. Baseline measurements were then conducted, consisting of at least three repetitions of cardiac index measurements by rebreathing (CI_rb_), FBP, HR and ICG in a standing position in the airplane cabin with the doors still open. The subjects were then seated for taxiing and take off for approximately 30 min. After a steady flight level was reached, three outbound data sets were collected in the standing position, and a second blood sample was obtained. During the flight phase of the parabolic trajectories, rebreathing exercises were performed in the standing position only at 0 and 1.8 G_z_ during parabolas 2–5 (block 1), 14–16 (block 2), 17–19 (block 3) and 27–30 (block 4). BP, HR and ICG data were collected continuously. Subjects stood in the upright body position during parabolas 1–6, 12–21 and 27–31 and were sitting during the remaining 10 parabolas (Fig. 1) to recover from the intense orthostatic challenge and thus to decrease their risk of presyncope and motion sickness. Two more blood samples were obtained after the 16th and 31st parabolas. At least three sets of rebreathing exercises and cardiovascular data points were collected during the return flight, and another three sets were collected after landing on the ground while the subjects were still in the airplane but with the doors open. The final blood sample was also collected on the ground. We adopted a rigorous rebreathing procedure that enabled us to measure two subjects at the same time (Online Resource 1). In particular, an operator indicated the breathing frequency and depth by moving his hand up and down, and both subjects triggered their breath cycles to the hand signals. During the parabolic trajectories, the rebreathing maneuvers were strictly aligned to the pilot’s announcements of trajectory: (1) “10 s” (2) “pull up” with increased G_z_ load of up to 1.8 G_z_; (3) “20”, “30”, and “40”, signifying the rising angle of attack of the airplane; (4) “injection”, with a rapid decrease of the G_z_ load to approximately 0.05 G_z_; and (5) “pull out”, with an increased G_z_ load of up to 1.8 G_z._ Each phase lasted approximately 20–25 s. Controlled rebreathing was initiated after the pilot’s announcement of “10 s” for the hyper-g measurements or at the pilot’s announcement of “40” for the 0 G_z_ measurements during the final pull-up seconds. Thus, the breaths relevant for CI_rb_ determination occurred during the 1.8 and 0 G_z_ phases, respectively, and the rebreathing maneuver was completed before injection and pull out, respectively.

#### Hypobaric chamber

The actual individual parabolic flight protocol for 11 of the 18 subjects was identical to that of the hypobaric chamber test. This comparison was performed to determine any potential effects of hypobaric hypoxia, restricted water intake in flight and changes in body position on the parameters of interest and to separate such effects from the effects of hyper- and microgravity. Tests were performed in the hypobaric chamber of the DLR Institute of Aerospace Medicine in Cologne, Germany, which has dimensions of 2.8 × 2 m and provides seats for six people. One subject was tested during each chamber run, supported by two operators in the chamber. The subjects received identical instructions before the chamber run and their parabolic flights. The chamber runs started at the same time as the actual parabolic flights and lasted as long as the individual flight day. The rebreathing and body position protocols were the same as those performed in flight. The subjects received an equal amount of subcutaneous scopolamine before their chamber runs, and the parabolic flight blood draw protocol was performed similarly. The chamber was depressurized to the actual inflight cabin pressure of that particular subject’s flight. De- and re-pressurization of the hypobaric chamber had exactly the same duration as in the actual flight of the subject. The other environmental conditions in the chamber were approximately 60 % humidity, an ambient temperature of approximately 23 °C, an illumination level of approximately 150 lux, a light color temperature of approximately 3,000 K, a noise level of approximately 70 dB due to airflow and no significant vibrations.

### Measurements

#### Inert gas rebreathing

Cardiac index (CI_rb_), stroke index (SI_rb_), oxygen consumption (VO_2_) and lung tissue volume (Vt) were determined by inert gas rebreathing (IGR) using an Innocor^®^ commercial inert gas rebreathing device (Innovision, Glamsbjerg, Denmark). Oxygen saturation (SO_2_) of the arterial blood was measured during each rebreathing at a fingertip. The subjects breathed ambient air through a face mask fitted around the nose and mouth. When a CI_rb_ measurement was required, the system switched to a closed rebreathing mode. A respiration bag was automatically filled with a gas mixture composed of 29.5 % O_2_ in N_2_, 0.5 % N_2_O (soluble tracer gas) and 0.1 % SF_6_ (non-soluble tracer gas). In our study, the volume of the respiration bag was approximately 40 % of the vital capacity of the subject. The pulmonary blood flow (PBF), which, in the absence of significant shunts, is equal to cardiac output, was calculated on the basis of the soluble tracer gas disappearance rate (N_2_O), the total volume of the system and the Bunsen solubility coefficient of the tracer gas in blood (Clemensen et al. 1994) (for details see Online Resource 2).

#### Cardiovascular parameters

Continuous beat-by-beat finger blood pressure was measured using a Finometer MIDI device [Finapres Medical Systems (FMS), Amsterdam, The Netherlands], which uses a photoplethysmographic technique based on the volume clamp method of the Czech physiologist J. Peňáz. The finger cuff was placed around the third finger of the left hand and the left hand was fixed by a bandage at the level of the fourth intercostal space at the assumed level of the heart. The mean arterial pressure was calculated from the systolic and diastolic finger blood pressures by the formula $\left[ {P_{\text{diast}} + \frac{{P_{\text{syst}} - P_{\text{diast}} }}{3}} \right] .$ The systemic vascular resistance was calculated by dividing the mean arterial pressure by CO_rb_ $\left[ {\frac{\text{MAP (mmHg)}}{{{\text{CO}}_{\text{rb}} \left( {L \times \min^{ - 1} } \right)}} = {\text{SVR}} \left( {\frac{\text{mmHg}}{\text{min} \times L}} \right)} \right]$. Finger blood pressure, ECG and thoracic impedance data were measured continuously during the rebreathing pre- and post-flight on the ground, during the rebreathing maneuvers in the outbound and return phases and during the entire phase of the parabolic trajectories. These data were stored at 2,000 Hz using ACQ*Knowledge* ^®^ 4.0 software (Biopac Systems Inc., Goleta, CA, USA) on a laptop (Dell Precision Workstation, Dell Inc., Round Rock, USA) for post-flight analyses. A solid-state hard drive was used for data storage to prevent automatic computer shutdown at 0 G_z_, which would be triggered by the laptop built-in free-fall sensor that would mistakenly indicate that the computer was falling down during the 0 G_z_ phases. Rebreathing maneuvers were subsequently identified from the thoracic impedance signal. The finger blood pressure and heart rate were averaged across the three relevant breaths required for the measurement of the SI_rb_, and these averages were then processed as single blood pressure and heart rate data points for further analysis (Limper et al. 2011).

#### Blood counts and intravascular volume

During parabolic flights and hypobaric chamber experiments, 10-ml serum and 6-ml EDTA blood samples were drawn. The overall amount of blood drawn during an experiment day was 80 ml. Intravascular volume on parabolic flight days and in the hypobaric chamber was determined using the Optimized Carbon Monoxide Rebreathing Technique (CORT) (Prommer and Schmidt 2007; Schmidt and Prommer 2005). In each case, 3-ml EDTA blood samples (*S*-*Monovette* ^*®*^, Sarstaedt AG & Co., Nümbrecht, Germany) were drawn from the antecubital vein via intravenous puncture (21 G Venofix^®^ Safety, B. Braun Melsungen AG, Melsungen, Germany). Each subject performed the carbon monoxide rebreathing procedure only once at the German Aerospace Center in Cologne, Germany with less than 2 months between the parabolic flights and hypobaric chamber runs (Online Resource 2). During the parabolic flights and the hypobaric chamber runs, blood samples were drawn in 2.0-ml EDTA tubes via the 16 G intravenous line in the right antecubital vein. Blood draws were performed five times per experiment day: pre, outbound, post 16 (meaning after the 16th parabola), post 31 (after the 31st parabola) and post. Identical labeling was used for the blood samples of both facilities. Blood samples were immediately refrigerated at 5 °C after collection. Blood count analyses were performed following landing at a local medical laboratory via a routine clinical method (Laboratoire d’Analyses Weckerle, Martignas-sur-Jalle, France). Analyses were performed twice, and the average results of the duplicates were used for intravascular volume calculations. After the hypobaric chamber experiments, blood counts were analyzed immediately at the laboratory of the German Aerospace Center in Cologne, Germany using the ABX Pentra 60 hematology analyzer (Horiba ABX SAS, Montpellier Cedex, France).

#### Biochemical analyses

Following collection, all samples were refrigerated at 5 °C until centrifugation at 1,500 rpm for 15 min at 4 °C. Plasma and serum were then transferred to 1.5-ml tubes, immediately frozen on dry ice and then kept at −80 °C. Blood osmolality and albumin, cortisol, aldosterone, CT-_pro_AVP, renin_active_ and NT-_pro_BNP concentrations were determined using standard methods by a commercial biomedical laboratory (MVZ Labor Dr. Quade und Kollegen, Cologne, Germany) within 3 months of blood sampling (for details, see Online Supplement 2).

#### Statistical analyses

We evaluated 631 inert gas rebreathing data sets and the same amount of simultaneously collected ECG and finger blood pressure data sets. In total, 374 data sets were collected during the parabolic flight days, whereas 257 data sets were collected during the hypobaric chamber tests; 128 and 66 inert gas rebreathing maneuvers were performed at 0 and 1.8 G_z_, respectively.

Analysis of variance (ANOVA) tests using a general linear model evaluated fixed effects of facility (parabolic flight vs. hypobaric chamber) and phase (“pre”; “outbound”; “block 1, block 2, block 3 and block4”; “return” and “post”, Fig. 1), and their interactions. Subject ID was used as a random factor to account for between-subject variability. Where fixed factors were significant, post hoc Tukey’s Honestly Significant Difference Test was employed to identify significant differences. For Phase, identical phase names were used for both parabolic flights and the hypobaric chamber to increase clarity. *P* = 0.05 was taken as the minimum level of significance. All statistical analyses were performed using STATISTICA 10 (StatSoft, Inc., Tulsa, OK, USA).

## Results

The results which are given in the following originate from a mixed-gender sample (Table 1).

### Cardiovascular parameters

**Fig. 2:**
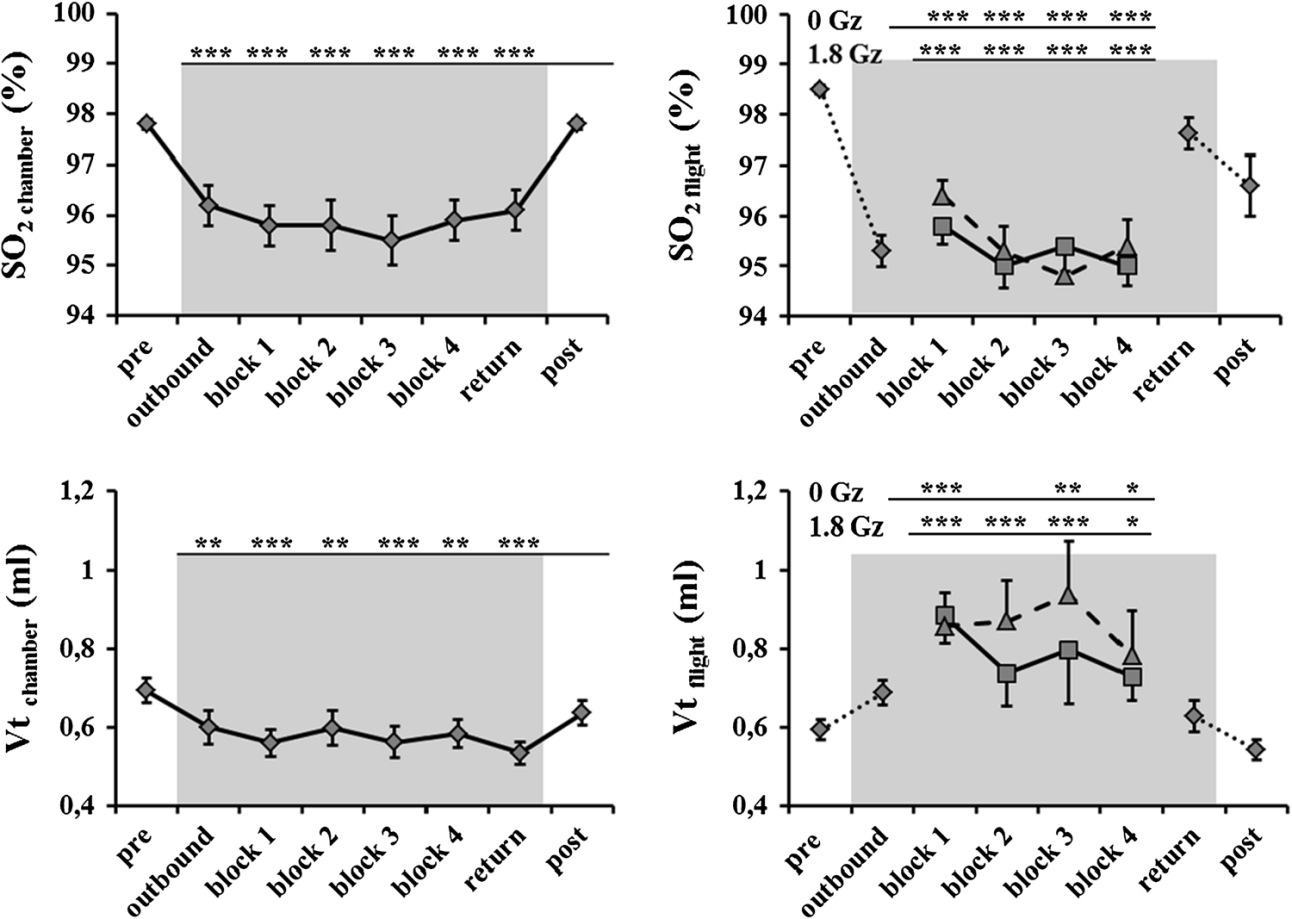
Time course of main pulmonary parameters in parabolic flight in 18 subjects and in hypobaric chamber in 11 subjects is shown as the mean ± SE;* asterisks *indicate significant differences with respect to pre: *p < 0.05, **p < 0.01, ***p < 0.001; *gray background* indicates measurements in hypobaric hypoxia after decompression to 830 mbar

**Fig. 3:**
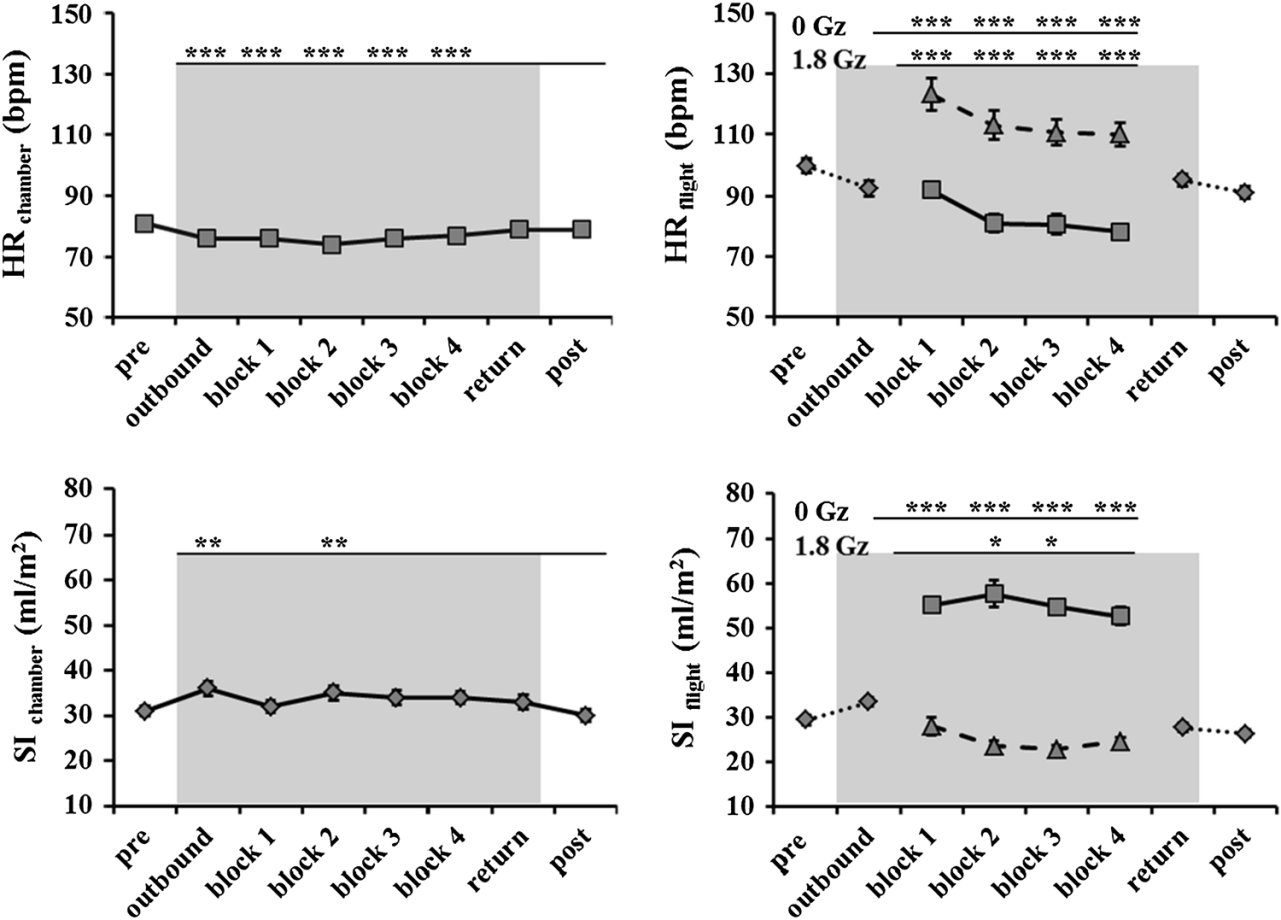
Time course of heart rate and stroke index responses in the hypobaric chamber and in parabolic flight; responses in 0 G_z_, *solid black* *graph* and at 1.8 G_z_;* dashed black graph* are shown separately. *Asterisks* indicate significant changes with respect to pre separately for hyper- and microgravity values; *gray background* indicates low ambient cabin pressure

**Fig. 4:**
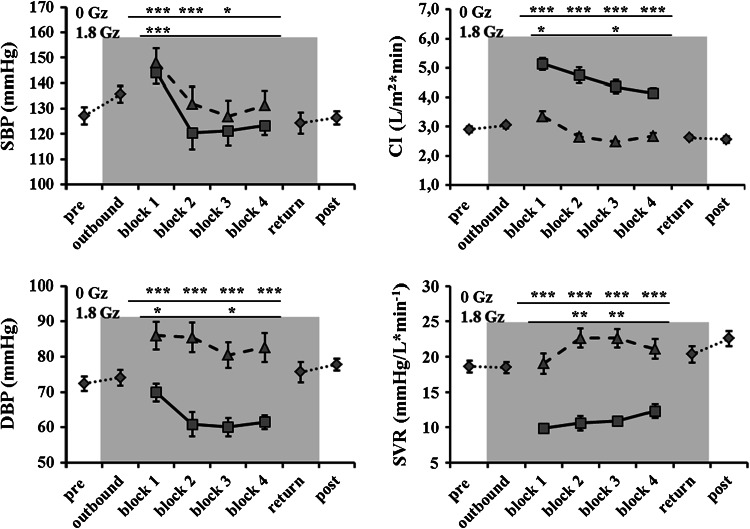
Time course of arterial pressure, systemic vascular resistance and cardiac index in parabolic flight; responses at 0 G_z_, *solid black* *graph* and at 1.8 G_z_, *dashed black graph*, are shown separately. *Asterisks* indicate significant changes with respect to pre separately for hyper- and microgravity values; *gray background* indicates low ambient cabin pressure

Figures 2, 3, 4 show the cardiovascular and pulmonary responses in parabolic flight and in the hypobaric chamber. HR decreased significantly during the hypobaric chamber run (*p* < 0.001) with respect to pre but did not differ during measurements post relative to pre (*p* = 0.639). In parabolic flight, HR was significantly decreased at 0 G_z_ with respect to pre (*p* < 0.001) and significantly increased at 1.8 G_z_ with respect to pre (*p* < 0.001). However, HR at 0 G_z_ decreased significantly over time (block 1 vs. block 4 *p* < 0.001). A similar attenuation in HR increase at 1.8 G_z_ was observed over time (block 1 vs. block 4, *p* < 0.001). HR was lower post-flight than in pre-flight measurements (p < 0.001).

The stroke index by rebreathing changed significantly during the hypobaric chamber run (*p* < 0.001) (Fig. 3, Online Supplements 3, 5). With respect to normobaric normoxia (NN) conditions at pre, SI_rb_ showed a significant increase during chamber outbound (*p* < 0.001) and block 2 (*p* < 0.001) and a tendency increase during block 3 and block 4 (*p* = 0.0775 and *p* = 0.0798, respectively). SI_rb_ was not significantly different between post measurements and pre measurements (*p* = 0.952). In parabolic flight, SI_rb_ was significantly enlarged at 0 G_z_ with respect to pre (*p* < 0.001). It was also significantly higher during outbound with respect to pre (*p* = 0.026). However, SI_rb_ did not decrease at 0 G_z_ over time (block 1 vs. block 4, *p* = 0.682). In the hyper-g of block 1, SI_rb_ showed no significant difference with respect to pre measurements (*p* = 0.999) but was subsequently smaller in block 2 and block 3 with respect to pre (*p* = 0.017 and 0.002, respectively). SI_rb_ was similar during pre- and post-flight measurements (*p* = 0.323).

With respect to pre baseline measurements, CI by rebreathing did not change significantly in any phase in the hypobaric chamber (Online Supplement 3, 5). However, in parabolic flight, CI_rb_ was significantly increased at 0 G_z_ with respect to pre in each block (*p* < 0.001). CI at 0 G_z_ decreased in a stepwise fashion (block 1 vs. block 4, *p* < 0.001). In block 1, CI_rb_ was significantly increased at 1.8 G_z_ with respect to pre, similar to pre in block 2 and block 4 (*p* = 0.660 and 0.817, respectively), and was significantly smaller with respect to pre measurements only in block 3 (*p* = 0.0252). Furthermore, CI_rb_ was significantly decreased after parabolic flight with respect to pre (*p* = 0.006).

The systolic blood pressure, shown in Fig. 4 and Online Supplements 3 and 5, did not show any significant change during the hypobaric chamber runs (*p* = 0.559). In contrast to the hypobaric chamber, FBP_syst_ changed significantly in parabolic flight, and the changes were of a similar pattern at 0 and 1.8 G_z_. FBP_syst_ was significantly increased in block 1 at both 1.8 and 0 G_z_ with respect to pre (*p* < 0.001 and < 0.001, respectively). During block 2 and block 3, FBP_syst_ was not different at 1.8 G_z_ but significantly decreased at 0 G_z_ with respect to pre (1.8 G_z_, *p* = 0.195 and 1.0, respectively; 0 G_z_, *p* < 0.001 and 0.007, respectively). During block 4, FBP_syst_ did not differ significantly at 0 G_z_ or at 1.8 G_z_ with respect to pre (*p* = 0.104 and 0.537, respectively). FBP_syst_ was similar after flight with respect to pre (*p* = 0.999).

Diastolic arterial pressure (FBP_diast_) did not show any change during the hypobaric chamber test (*p* = 0.814), as shown in the Online Supplements 3 and 5. In parabolic flight, FBP_diast_ was significantly increased at 1.8 G_z_ in each block with respect to pre (*p* < 0.001 each), as shown in Fig. 4. At 0 G_z_ during block 1, FBP_diast_ did not differ significantly from pre values (*p* = 0.102) but was significantly lower at 0 G_z_ in block 2 to block 4 with respect to pre (*p* < 0.001 each). After parabolic flight, FBP_diast_ remained higher than before parabolic flight (*p* < 0.001).

Systemic vascular resistance (SVR) did not change at any time in the hypobaric chamber (*p* = 0.921) (Online Supplements 3 and 5). However, in parabolic flight (Fig. 3), SVR showed a significant decrease at 0 G_z_ from block 1 with respect to pre (*p* < 0.001 for each block). At 1.8 G_z_ of block 1, SVR was statistically similar to pre (*p* = 0.999) but increased compared to pre values, in block 2 and block 3 (*p* < 0.001 each). In block 4, SVR was again not significantly different from pre values (*p* = 0.198). However, SVR was significantly increased after parabolic flight with respect to pre values (*p* < 0.001).

### Pulmonary parameters

Oxygen saturation was significantly decreased in reduced ambient pressure with respect to regular ambient pressure in both parabolic flight and in the hypobaric chamber (*p* < 0.001 and *p* < 0.001, respectively) (Fig. 2 and Online Supplements 4 and 5). With respect to normobaric normoxia baseline measurements, pulmonary tissue volume was significantly decreased only in hypobaric hypoxia conditions in the hypobaric chamber (*p* < 0.001); in contrast, it was significantly increased at 0 and 1.8 G_z_ (*p* < 0.001 and *p* < 0.001) (Fig. 2 and Online Supplements 4 and 5). Oxygen consumption did not show any significant change in the hypobaric chamber in HH relative to regular pressure (*p* = 0.330). Oxygen consumption was not different between 1.8 G_z_ and baseline 1 G_z_ + NN but was significantly increased at 0 G_z_ relative to 1 G_z_ + NN (*p* < 0.001) (Online Supplements 4 and 5).

### Plasma volume

**Fig. 5:**
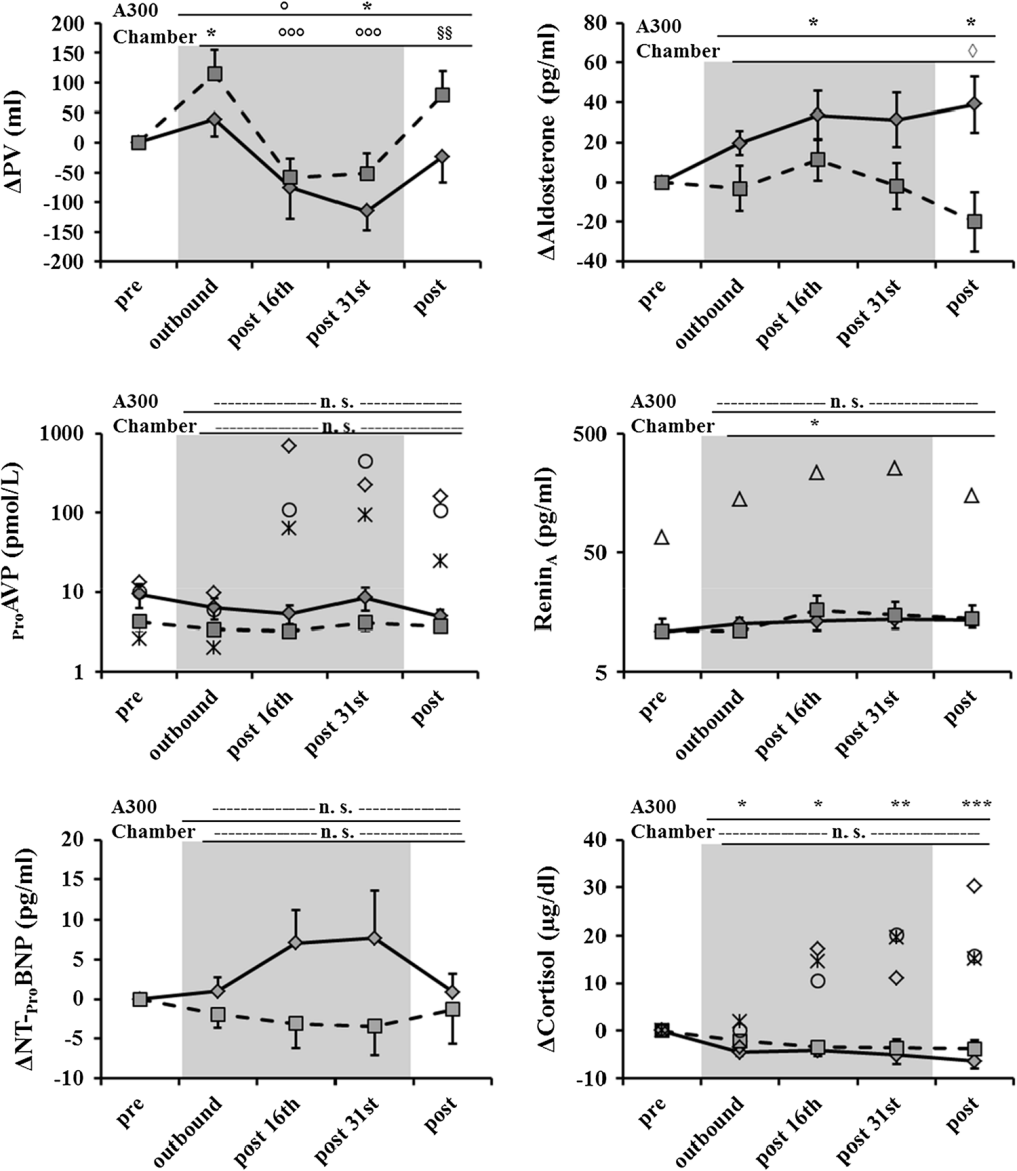
Courses of plasma volume and a subset of blood hormones are shown as a continuous *black graph* for parabolic flight and as *dashed black graph* for the hypobaric chamber results; significant differences with respect to pre are indicated as *asterisk* in parabolic flight (A300) and in the hypobaric chamber (chamber); the statistical significance of plasma volume and ΔAldosterone is illustrated as *asterisk* with respect to pre; *degree symbol* with respect to outbound; *open diamond* with respect to post 16th; and* section symbol* with respect to post 31st. In the Δ_pro_AVP and Δcortisol diagrams, the *black open circle* represents subject 0AP, the *black cross* represents subject 0AD, the *black open* diamond represents subject 0AT; in the renin_active_ diagram (Renin_A_), the *black open triangle* represents the individual responses of subject 0AL; *gray background* indicates hypobaric hypoxic conditions

Figure 5 shows that the response patterns of plasma volume did not differ significantly between parabolic flight and the hypobaric chamber (*p* = 0.449). However, responses in the hypobaric chamber seemed to be more distinct. In parabolic flight, there was a significant decrease in plasma volume after parabola 31 with respect to pre (*p* = 0.034). Pre and post values were not significantly different on the parabolic flight day (*p* = 0.9728). In the hypobaric chamber there was a significant increase in plasma volume during outbound with respect to pre (0.024) and then a significant decrease in plasma volume at post 16 and post 31 with respect to outbound (*p* < 0.001 and <0.001, respectively). Post-flight plasma volume recovered to baseline values again and was significantly higher than at post 16 and post 31 (*p* = 0.004 and 0.007, respectively) (Online Supplement 6).

### Blood hormones and osmolality

Cortisol and CT-_pro_AVP are strongly influenced by motion sickness (Schneider et al. 2007; Kohl 1987; Drummer et al. 1990). Thus, the three motion-sick subjects 0AD, 0AP and 0AT were excluded from the statistical analyses of cortisol and the CT-_pro_AVP parabolic flight values, but their responses are shown individually in Fig. 5. Subject 0AL showed a very different renin response pattern in the hypobaric chamber; thus, the renin hypobaric chamber values of 0AL were removed from the statistical analysis but are provided individually in Fig. 5. Depending on the inter-individual variance, some parameters are shown as differences from the baseline and some as absolute values.

ΔCortisol decreased significantly after the parabolic flight relative to pre-flight measurements (*p* < 0.001). In the hypobaric chamber, a significant change in cortisol over time was also visible (*p* = 0.0475). CT-_pro_AVP did not show any difference between its response to parabolic flight and its response to the hypobaric chamber (*p* = 0.810). In both facilities, CT-_pro_AVP was not different after the run with respect to baseline levels (*p* = 0.154 and 0.566). Similarly, there was no significant difference in the course and absolute values of osmolality between both facilities (*p* = 0.726 and *p* = 0.379, respectively), although osmolality appeared to be slightly higher on parabolic flight days. Renin_active_ did not react differently between the hypobaric chamber and parabolic flight (*p* = 0.213). However, post 16th renin showed a significant increase with respect to pre (*p* = 0.013). In parabolic flight, renin_active_ did not change significantly over time (*p* = 0.168). The time course of aldosterone levels was significantly different between the hypobaric chamber and parabolic flight (*p* = 0.044). In the hypobaric chamber, aldosterone levels tended to decrease over time with respect to pre without reaching statistical significance (*p* = 0.0926), but the decrease was significant after the chamber run with respect to post 16th (*p* = 0.0458). In parabolic flight, aldosterone levels increased over time. At post 16th and post-flight, aldosterone levels were significantly elevated with respect to pre (*p* = 0.041 and 0.011, respectively). The time course of NT-_pro_BNP was statistically not different between the hypobaric chamber and parabolic flight (*p* = 0.136). However, it showed a strong tendency to increase in the parabolic flight (*p* = 0.075) and to decrease in the hypobaric chamber (*p* = 0.056) (Online Supplement 6).

## Discussion

The major findings of the study are fourfold. *First*, confirmation of the observations of the studies of Iwase et al. (1999a) and Beckers et al. (2003) who found that cardiovascular responses to the transition into weightlessness in a standing position are different between the early parabolas of a parabolic flight and the later parabolic phases. Our results give new insights into the mechanisms of those differences by showing that, in the early phase of the flight, there is no distinct blood pressure decrease after the injection of 0 G_z_, which would have been expected. However, SVR is decreased at 0 G_z_ from the early phase of the flight on. Thus, blood pressure is kept elevated by a lack of heart rate decrease after injection together with an already increased stroke volume. Over the course of a parabolic flight day, the heart rate at 0 G_z_ decreases and through decreased cardiac output leads to a pronounced blood pressure drop at 0 G_z_ in the later phases. *Second*, the plasma volume response pattern in the hypobaric chamber and during parabolic flight is comparable. With respect to the baseline, an increase in PV during outbound was observed in both facilities, followed by a decrease after the 16th and 31st parabola and an increase after recompression. This pattern suggests that changes in plasma volume depend mainly on changes in body position and changes in ambient and oxygen partial pressure and depend to a lesser degree on gravity changes produced by parabolic trajectories. *Third*, differences in hormonal responses occur between the two facilities. Whereas the combination of hypobaric hypoxia and gravity changes in parabolic flight induces an increase in aldosterone, the opposite is the case after the hypobaric chamber run. NT-_pro_BNP shows a strong tendency to increase during parabolic flight maneuvers but not in hypobaric hypoxia in a hypobaric chamber alone. Renin_active_ is increased in the hypobaric chamber but is not affected by parabolic flight. *Fourth*, the lungs and the cardiovascular system interact differently in the two facilities. Whereas in the hypobaric chamber, lung tissue volume is decreased by hypoxic pulmonary vasoconstriction, lung tissue volume is increased at 0 and in 1.8 G_z_ in parabolic flight.

Our results show that the cardiovascular response to microgravity transitions in the standing position differs between the early and later parabolas. This finding is of importance for the design and the interpretation of cardiovascular experiments on parabolic flights. Our results suggest that data collected during the initial five parabolas of a parabolic flight should be discarded to increase the homogeneity of the cardiovascular results. The observed variability may be partly derived from the administration of scopolamine, which has a serum elimination half-life of approximately 2 h (Stetina et al. 2005). This point may suggest that half-elimination of scopolamine from the circulation has already occurred after an early parabolic flight phase. However, the increased level of psychomotor excitement is also certainly of importance. In contrast, changing interactions of the baroreflex and a “Bainbridge-like Reflex” may explain changes in cardiovascular responses over the parabolic flight. Whereas the Bainbridge-like reflex induces tachycardia triggered by a central volume increase by a volume shift at 0 G_z_ (Petersen et al. 2011), the baroreflex would induce bradycardia. It is possible that the Bainbridge-like reflex predominates in early parabolic flight because the vagotropic blocking capability of Scopolamine suppresses the vagal efferent outflow of the baroreflex. The sympathetic withdrawal effect of the baroreflex at 0 G_z_ seems to be unaffected, as indicated by a decreased SVR at 0 G_z_ from the very first parabola (Fig. 3) and as found by Iwase et al. (1999b). However, whether factors other than scopolamine, e. g., hypobaric hypoxia, influence arterial and cardiac regulation remains unclear. Systolic and diastolic blood pressure was unaffected in the hypobaric chamber, whereas heart rate decreased, which could indicate a resetting of the baroreflex. However, the neutral, rather tedious environment of the confined hypobaric chamber experiment could well have contributed a further decrease in heart rate. How the baroreflex behaves in hypoxia and hypobaric pressure has been inconsistently defined in the literature. Halliwill and Minson (2002) present data supporting that hypoxia resets the baroreflex and muscle sympathetic nerve activity (MSNA) to higher levels without changing the baroreflex sensitivity, whereas Sevre et al. (2002) found evidence for reduced baroreflex sensitivity in a hypobaric chamber experiment simulating an airplane cabin atmosphere with a pressure equivalent to an altitude of 2,400 m (Sevre et al. 2002; Halliwill and Minson 2002). Finally, heart rate increases typically in hypoxia caused by decreased oxygen partial pressure in the blood (West et al. 2007), which was contrary to the findings of our hypobaric chamber runs.

We found a slight intermittent decrease in plasma volume over both parabolic flight and hypobaric chamber courses. This finding was contrary to our expectations. Schlegel reported that the 16 subjects of the parabolic flight experiment had on average a larger stroke volume in the supine position when compared between and after the flight, suggesting an increase in blood volume (Schlegel et al. 2001). During the parabolas, the subjects had been in an upright sitting position, which allowed a certain value of volume shift through gravity transitions. Schlegel did not measure intravascular volume and hormone concentrations directly but actually focused on the question of whether changing levels of arginine vasopressin (AVP) and renin–angiotensin–aldosterone could lead to an increase in intravascular volume during parabolic flights. Schlegel suggested, based on previous work, that the predominance of hyper G_z_ during the flight with respect to μG_z_ may have led to the expansion of intravascular volume. Indeed, the overall durations of the μG_z_ and hyper G_z_ phases during a parabolic flight were approximately 1,000 and 2,000 s, respectively, on the KC 135 and approximately 700 and 1,400 s, respectively, on the A300 Zero-G, and therefore twice as long under hyper than under μG_z_. Those longer micro- and hypergravity phases and the different flight profile on KC 135 parabolic flights may be an explanation for the different findings between Schlegel’s and our work. Nevertheless, we found, in accordance with the concept of a modification of the Starling–Landis pressure under changing gravity as noted by Hargens and Richardson (2009), a plasma volume loss during parabolic flight and a recovery to baseline values after re-pressurization. Again, we would have expected that such a contraction of intravascular volume through the effects of hyper G_z_ and μG_z_ on the Starling–Landis equation during the parabolic flight day would have been aggravated by hemoconcentration because of the hypobaric hypoxia of the airplane cabin inflight. It is well known that hypoxia induces a reduction in plasma volume that increases the hematocrit and thereby improves tissue oxygenation (Bartsch and Saltin 2008). This finding is already accounted for from just slight hypobaric hypoxia, i.e., in an ambient pressure equivalent to an airplane cabin inflight. However, in contrast to our expectation, Yamashita et al. were not able to find a significant effect of 130 min of quiet sitting in a hypobaric chamber in an ambient pressure equivalent to the pressure at 2,000 m and a low relative humidity of 20 % on hematocrit levels with respect to the regular ambient pressure of sea level (Yamashita et al. 2005). However, they did find a significant decrease of body weight (100–200 g) after the chamber test with respect to baseline values. This finding may indicate a loss of extracellular water with a concomitant preservation of intravascular volume. Yamashita et al. concluded that low humidity conditions may have a higher effect on fluid loss than the hypobaric hypoxia itself. However, we found more pronounced changes in plasma volume over the course of the hypobaric chamber testing than during the parabolic flight, whereas the response pattern for the plasma volume in both facilities seemed to be similar. An explanation for the greater plasma volume changes in the hypobaric chamber, in addition to the smaller subject collective, may involve a different volume status of the subjects during the chamber runs as indicated by a slightly lower average osmolality during the chamber runs. The high average osmolality of approximately 310 mosmol/kg in the subjects during the parabolic flights is a strong indicator for a dehydrated state on the flight days. AVP is known to be increased in volume-contracted subjects, and CT-_pro_AVP shows a similar pattern for changes in blood volume (Szinnai et al. 2007). The reduced fluid volume in parabolic flight may be explained by the participants’ overnight fasting and then only having a slight breakfast without much morning fluid intake. The lower humidity in the airplane cabin inflight with respect to the chamber may have been an additional factor in the differences in osmolality, but the difference was already apparent during the baseline measurements. However, it should be taken into account that high osmolality has an impact on cardiovascular reflexes. Charkoudian et al. reported that hyperosmolality of even 290 mosmol/kg increases the baroreflex sensitivity in young subjects and has a sympathoexcitatory influence in general (Charkoudian et al. 2005).

In the present study, hormones related to volume regulation and their precursor peptides responded differently to scopolamine, standing position, gravity changes and HH in the airplane cabin on the one hand and to scopolamine, standing position and HH in the hypobaric chamber on the other hand.

Whereas aldosterone values increased in HH in combination with changing g-loads during the parabolic flight, they decreased in HH in the hypobaric chamber. Aldosterone levels are expected to decrease after a move to higher altitude and in hypoxia (Slater et al. 1969; Shigeoka et al. 1985). However, an aldosterone increase at altitude was reported by Humperler et al. (1980) and attributed by Richalet (2001) to the physical exercise in the study. Under orthostatic stress, aldosterone levels are known to increase (Laszlo et al. 2001). Taken together, these studies underpin the following interpretation of our own results: in the hypobaric chamber, the serum aldosterone concentration decreased in response to HH. In contrast in parabolic flight, increased orthostatic stress and muscular load of standing upright during hyper-g phases, and increased postural muscular work due to turbulent flight phases, and potentially increased muscular work, induced by the airplane’s whole-body vibrations, increased aldosterone release. This finding could be interpreted as supporting the effects of orthostatic and exercise stress on the effect of the stress of HH during parabolic flight on aldosterone release.

Indeed, renin_active_ did not show a parallel response with aldosterone. The renin_active_ post 16th measurement during hypobaric chamber testing showed a slightly significant increase with respect to pre. However, renin_active_ did not show any further significant response, neither in parabolic flight nor in hypobaric chamber. This implies a dissociation of the aldosterone level and renin response under the particular conditions of parabolic flight. Interestingly, dissociation of plasma aldosterone levels and plasma renin activity has previously been reported in subjects experiencing presyncope and in subjects undergoing repeated orthostatic challenges by tilt table testing and lower body negative pressure (LBNP) tests (Roessler et al. 2011; Hinghofer-Szalkay et al. 2011). The works of Roessler et al. and Hinghofer-Szalkay et al. indicate furthermore that during orthostatic challenge aldosterone is rather controlled by adrenocorticotropic hormone (ACTH) than by renin, which may serve as an explanation of the dissociated aldosterone–renin response in parabolic flight.

NT-_pro_BNP shows a strong tendency toward a different response in the chamber with respect to parabolic flight (*p* = 0.0524). Parabolic flight induces an increase, whereas only hypobaric hypoxia and a standing position do not affect NT-_pro_BNP. This finding is in agreement with the literature, which reports that NT-_pro_BNP does not increase with an acute ascent to altitude (Toshner et al. 2008) and during tilt table orthostatic tests, but does increase after an increase in thoracic blood volume-by-volume loading in healthy volunteers (Heringlake et al. 2004). This finding may indicate that the trend toward increased NT-_pro_BNP during parabolic flight is triggered by reiterative increasing in the thoracic blood volume at 0 G_z_. With subjects 0AT, 0AP and 0AD excluded from the analysis of the parabolic flight data for cortisol because of possible stress responses arising from motion sickness, cortisol showed a significant decrease over the day in both facilities. This finding underpins the observation of a decreased heart rate during the flights and shows that the stress level decreases. However, this response results not only from decreasing excitement during the experiments but also from the distinct circadian rhythm of cortisol. The blood cortisol concentration shows a physiological peak between 8 and 9 a.m. and a continuous decrease thereafter. Peak values in healthy subjects are approximately 16 μg/dl and decrease to approximately 12 μg/dl at noon (Debono et al. 2009). The baseline measurements before the flights and the chamber runs were obtained between 8 and 9 a.m., i.e., during the physiological cortisol peak, and post-intervention measurements were obtained between noon and 1 p.m. The measured cortisol values at these time points were similar to the circadian values of these day phases given in the literature by Debono et al. (2009)

Opposing alterations of pulmonary tissue volume in hypergravity and microgravity compared with hypoxia have been shown in different experiments. Snyder et al. (2006) showed a decrease in lung water and lung tissue volume under moderate hypoxia of 12.5 % inspired oxygen in resting healthy subjects. Rohdin and Linnarson found increased lung tissue volume in healthy subjects during 2 and 3 G_z_ centrifugation in a sitting position. Furthermore, they reanalyzed the parabolic flight data of Vaida et al. and noted an increase in lung tissue volume in weightlessness (Vaida et al. 1997; Rohdin and Linnarsson 2002). Vaida had performed the experiment during parabolic flights in the former European Caravel parabolic flight airplane under a hypobaric ambient pressure of 793 mbar. Thus, the results of Snyder, Rohdin and Vaida are in line with our observations of decreased Vt in the hypobaric chamber and increased Vt during weightlessness and hypergravity in parabolic flight. However, we are the first to show in the same subjects that the reduction in blood volume in hypobaric hypoxia of the airplane cabin is reversed by a central volume shift in weightlessness and by sequestration, as suggested by Rohdin and Linnarsson, of blood in the dependent parts of the lung circulation in the hypergravity phases. This finding could be of benefit for potential parabolic flight candidates suffering from pulmonary hypertension, which would be aggravated by the hypobaric hypoxia of the airplane cabin and which may be attenuated by the pulmonary response to hyper- and microgravity.

## Limitations

The study design included some limitations that we tried to consider in our interpretation of the results. *First*, temperature, humidity, noise level, vibrations and light conditions in the hypobaric chamber were not fully comparable to parabolic flight because of a lack of air-conditioning in the hypobaric chamber, and because of a fixed installed non-changeable illumination system in the hypobaric chamber. It seems unlikely that the slightly higher temperature in the hypobaric chamber of approximately 23 °C, with respect to approximately 19 °C in the cabin of the A300 inflight, led to changes in the orthostatic or volume-regulating behavior of the cardiovascular system for instance by skin vasodilation or even by increase of the core body temperature (Allan and Crossley 1972). There was a 10 dB difference in the noise level during parabolic flight with respect to hypobaric chamber runs. Thus, in both facilities the noise level was comparable and below 90 dB which is known to increase the degree of physiological arousal (Harding and Mills 1983) and therefore we do not assume a significant effect of differences in the noise levels of the two facilities on our data. On the other hand, vibrations which appeared only inflight and not in hypobaric chamber might have had a certain minor impact on our results. Although exposure to moderate levels of whole-body vibrations does not lead to consistent changes in basic measures of the cardiovascular system, there may be an increase in muscle activity to maintain body posture which may again lead to peripheral vasoconstriction (Rollin Stott 2006). Furthermore, whole-body vibration induces a slight increase in metabolic rate which is comparable with that seen in light exercise and to hyperventilation with a reduction in CO_2_ (Rollin Stott 2006). However, forced hyperventilation due to vibrations inflight, with respect to the hypobaric chamber, would have led to an increase in SO_2_ of the arterial blood inflight with respect to SO_2_ in the hypobaric chamber, which indeed cannot be found in our data. The differences in illumination between the hypobaric chamber and the cabin of the A300 were approximately 650 lux in brightness and 600 K in light color temperature. Noguchi investigated the influence of 50 and 150 lux of light brightness and 3,000 and 5,000 K of light color temperature on the activity of the autonomic nervous system. They could not find any difference in the activity of the autonomous nervous system under these conditions what makes us doubting a significant effect of the differences in light characteristics in our study on our data (Noguchi and Sakaguchi 1999). *Second*, it is known that vestibular–autonomic interactions (Yates 1996) and cardio-postural interactions (Goswami et al. 2012; Blaber et al. 2009) affect cardiovascular responses during orthostatic stress. Therefore, subjects were instructed to avoid head movements during the hypergravity phases to minimize potential vestibular–autonomic interactions. However, minor differences in cardio-postural interactions in parabolic flight with respect to hypobaric chamber can be considered possible because in parabolic flight subjects were standing on a floor covered with soft padding and trying to adjust their upright body posture for turbulences using their postural muscles. Furthermore, large muscle groups may have been activated by airplane whole-body vibrations during flight. These advanced postural adjustments, which almost did not occur in hypobaric chamber, may have led to increased muscle pumping and increased venous return inflight with respect to the hypobaric chamber. *Third*, only 11 of the 18 subjects of the parabolic flights were available to participate thereafter in the hypobaric chamber tests*. Fourth*, three of the 18 subjects developed motion sickness in parabolic flight, which affects cardiovascular and hormonal regulation and removes the homogeneity of the subject population. It is well known that levels of AVP and cortisol are extensively increased in motion sickness; therefore, the CT-_pro_AVP and cortisol values of the motion-sick subjects were excluded from the statistical analysis of the blood hormones, and individual data are shown instead. Furthermore, we did not analyze blood levels of ACTH what might had allowed us to identify a close relationship between ACTH and aldosterone in parabolic flight as it is known for orthostatic stress during tilting and LBNP (Roessler et al. 2011; Hinghofer-Szalkay et al. 2011). *Fifth*, rebreathings at 0 G_z_ fell into the early 0 G_z_ phases, which are characterized by a sympathetic withdrawal and acute activation of the vagal nervous system. Later in the 0 G_z_ phase, there would be an increasing dominance of the sympathetic nervous system. We did not perform most of the rebreathings in this phase, and thus our results mainly represent the cardiovascular responses in the early 0 G_z_ phases. *Sixth*, forced breathing, as performed for the rebreathing maneuvers for CI_rb_ determination, modulates cardiovascular regulation during gravity transitions (Schlegel et al. 1998; Iwase et al. 1999b). However, using a breathing frequency of 20 breaths per minute and a rebreathing volume between 1.5 and 2.5 L, we were in the range of the low effect of breathing parameters on CO_rb_ influencing noted by Damgaard and Norsk (2005).

## Conclusion

In conclusion, the cardiovascular, pulmonary and body fluid system are influenced not only by micro- and hypergravity but also by the hypobaric hypoxic cabin environment of the parabolic flight airplane. This finding leads, in some cases, to antagonistic reflex patterns in which reflexes triggered by GT abolish those triggered by HH. The compensation of the hypoxic pulmonary vasoconstriction by volume shift and the increases in cardiac output during parabolic flight maneuvers could have a positive effect on some potential parabolic flight participants with restricted health status, e.g., patients with mild chronic obstructive pulmonary disease or right ventricular strain; these effects should be investigated in future studies.

## Electronic supplementary material

Below is the link to the electronic supplementary material.

**Online Resource 1.:** The video shows the five members of the experiment team inflight on board A300 Zero-G during a single parabola. The pilot’s announcements of the parabolic phases from the cockpit are audible. The experiment rack consists of two identical test units including in duplicate: Innocor rebreathing device; Biopac device for ECG, ICG and accelerometer data collection; Finometer MIDI non-invasive finger blood pressure measurement device; laptop for data storage and a battery of blood sampling tubes. The cooler for blood sample storage is not visible on the video. The experiment design allows the simultaneous accomplishment of multiple measurements in two subjects. The two subjects are connected via face masks to the two Innocor rebreathing devices and via an “umbilical” cord to the Finometer and Biopac units. Two operators fixed to the floor by straps and harnesses are assisting the subjects during the hyper-g phases and especially during free floating passively after the transition into weightlessness. A third operator is leading the subjects through the rebreathing procedure by guiding the subjects’ breathing with hand signs and starting the measurements immediately after the transition to weightlessness. (MPG 263130 kb)

**Online Resource 2.:** Extended methodological description including additional fundamental literature for the procedures of inert gas rebreathing, intravascular volume determination and biochemical analyses. (DOCX 33 kb)

**Online Resource 3.:** Cardiovascular results of N = 18 as the mean ± SD of the parabolic flight tests are shown. (DOCX 16 kb)

**Online Resource 4.:** Pulmonary results of N = 18 as the mean ± SD of the parabolic flight tests are shown. (DOCX 15 kb)

**Online Resource 5.:** Cardiovascular results as the mean ± SD of the hypobaric chamber tests are shown. N = 11 for both pulmonary and cardiovascular parameters. (DOCX 16 kb)

**Online Resource 6.:** Plasma volume and biochemical responses in parabolic flight and the hypobaric chamber are given as the mean ± SD; # indicates parabolic flight values as the mean ± SD, which are presenting excluding the responses of the motion-sick subjects 0AP, 0AD and 0AT. The parabolic flight values of these subjects’ parameters are shown individually in Fig. 4. ‡, renin_active_ responses of subject 0AL in the hypobaric chamber were not included for mean and SD calculations because of an exceptional response. The 0AL-renin_active_ responses are shown individually in Fig. 4. (DOCX 16 kb)

## Abbreviations

ACTH: Adrenocorticotropic hormone
BMI: Body mass index
CI: Cardiac index
CI_rb_: Cardiac index by rebreathing
CO: Cardiac output
CO_rb_: Cardiac output by rebreathing
CORT: Optimized carbon monoxide rebreathing technique
dB: Decibel
DLR: German Aerospace Center
FBP: Finger blood pressure
FBP_diast_: Diastolic finger blood pressure
FBP_syst_: Systolic finger blood pressure
GT: Gravity transition
G_z_: Gravity load in head to toe direction
HH: Hypobaric hypoxia
HPV: Hypoxic pulmonary vasoconstriction
HR: Heart rate
ICG: Impedance cardiography
IGR: Inert gas rebreathing
LBNP: Lower body negative pressure
m. a. s. l.: Meters above sea level
MSNA: Muscle sympathetic nerve activity
NN: Normobaric normoxia
PBF: Pulmonary blood flow
PV: Plasma volume
SI: Stroke index
SI_rb_: Stroke index by rebreathing
SO_2_: Arterial oxygen saturation
SV: Stroke volume
SV_rb_: Stroke volume by rebreathing
VO_2_: Alveolar oxygen consumption
Vt: Volume of pulmonary tissue
