# Supplementary material for: Interactions of the human cardiopulmonary, hormonal and body fluid systems in parabolic flight
Source: Eur J Appl Physiol. 2014 Mar 13;114(6):1281–95. doi: 10.1007/s00421-014-2856-3 (PMC4019836; doi:10.1007/s00421-014-2856-3)
Supplement: Supplementary file 2 — Online Resource 2.: Extended methodological description including additional fundamental literature for the procedures of inert gas rebreathing, intravascular volume determination and biochemical analyses. (DOCX 33 kb) [file 421_2014_2856_MOESM2_ESM.docx]

*Cardiac Output by Inert Gas Rebreathing*

In this study, the test subjects performed the rebreathing procedure with a respiratory rate of 20 breaths per minute in around 15 seconds (Online Resource 1). Thus, rebreathing maneuvers lay in the limits with least effects on CO, as postulated by Damgaard and Norsk ([Damgaard and Norsk 2005](#_ENREF_4)). Stroke volume (SV_rb_) was calculated afterwards by dividing pulmonary blood flow (PBF) by heart rate (HR) from ECG. HR determined by Innocor from finger pulse oxymeter plethysmogram could not be used, because Innocor uses the average HR value taken from a 30 seconds interval just before the actual rebreathing (personal communication of Innovision). Cardiac index (CI_rb_) and stroke index (SI_rb_) were then obtained by dividing CO_rb_ and SV_rb_ by body surface area which was calculated automatically by the Innocor by the formula of Du Bois and Du Bois ([Du Bois and Du Bois 1916](#_ENREF_5)). Combined lung tissue volume and capillary blood volume (Vti,c), hence the volume of solvent, is calculated by the Innocor device on the basis of the initial fall in N_2_O concentration ([Clemensen et al. 1994](#_ENREF_3)). Therefore it is assumed that the volume of N_2_O absorbed by the lung is measured during midinspiratory time of the first inhalation from the bag before the gas is taken up in significant amounts by the capillary blood flow ([Clemensen et al. 1994](#_ENREF_3); [Sackner et al. 1964](#_ENREF_10); [Cander and Forster 1959](#_ENREF_2)). Alveolar oxygen consumption (VO_2_) was determined by Innocor just during rebreathings on the assumption that the slope of the disappearance curve for O2 is proportional to the oxygen uptake ([Clemensen et al. 1994](#_ENREF_3); [Lang et al. 2007](#_ENREF_7)).

*Intravascular Volume*

The determination of intravascular volume is based on a carbon monoxide dilution technique (CORT) and the determination of the total hemoglobin (Hb) mass. Measurements of the carboxyhemoglobin fraction were performed before and after the carbon monoxide rebreathings via the Radiometer ABL 725 blood gas analyzer (Diamond Diagnostics, MA, USA) which has been proven as a feasible device for carboxyhemoglobin determination ([Andersson and Moller 2010](#_ENREF_1)). A maximal 2 month distance between the CORT procedure and the experiments in the flights and the hypobaric chamber seemed feasible because the individual total Hb mass is very stable over time. Eastwood and colleagues found a variation in total Hb mass of less than 2% over 100 days ([Eastwood et al. 2008](#_ENREF_6)). By assuming the same total hemoglobin mass during the flights and hypobaric chamber runs as during the actual rebreathing procedure intravascular volumes could be calculated from venous blood counts which were drawn during the flights and the chamber runs. The actual total hemoglobin mass was corrected, considering Hb mass loss due to the 16 ml of blood drawn at each sampling, by using the equation $\left[ tHb\left( g \right)-\left( 16\left( ml \right)*Hb\left( \frac{g}{dl} \right) \right) \right]$.

*Biochemical Analyzes*

Blood osmolality was determined in serum by the freezing point depression method (OM 801, Vogel GmbH, Giessen, Germany). 280–300 mosmol/kg was given by the executing laboratory as normal range. Albumin was determined in blood serum by a BCG dye binding assay (ADVIA 1800, ADVIA Chemistry Systems, Siemens Health Care Diagnostics GmbH, Eschborn, Germany). Albumin normal range was stated as 35.0 – 52.0 g/L. Blood cortisol concentration was determined from serum by an ELISA assay with 4.3 – 22.4 µg/dl as normal range (Siemens ADVIA Centaur Cortisol, Siemens AG, Munich, Germany). Direct renin activity (DRA) measurement was performed in EDTA plasma by a chemiluminescence immuno assay (LIAISON Direct Renin, DiaSorin S.p.A., Saluggia, Italy) ([Rossi et al. 2010](#_ENREF_9)). Normal range for DRA was set for upright body position at 2.46 – 25.8 pg/ml. Blood aldosterone concentrations were measured in serum by an ELISA assay (DB 52001, IBL International GmbH, Hamburg, Germany). Aldosterone normal range in upright body position was given as 40.0 – 310.0 pg/ml. To measure the behavior of arginine vasopressin (AVP) during the parabolic flight and hypobaric chamber tests we determined the concentration of the C-terminal part of the AVP precursor (copeptin) in the blood serum via an immunofluorescent assay (B·R·A·H·M·S Copeptin us KRYPTOR, B·R·A·H·M·S GmbH, Hennigsdorf, Germany). The normal range of copeptin is dependent on the blood osmolality. For a blood osmolality between 291 and 300 mosmol/kg the normal range of copeptin is 2.3 – 28.2 pmol/L. Morgenthaler et al. pointed out the high stability of copeptin in serum what mature AVP unlike is and which can be taken as the most relevant advantage of the copeptin assay ([Morgenthaler et al. 2008](#_ENREF_8)). NT-proBNP, as a stable surrogate of BNP, was determined by a chemiluminescent immunometric assay in EDTA blood plasma (IMMULITE 2000 systems, Siemens AG, Munich, Germany). Cut off value for heart failure was set by the laboratory at 125 pg/ml.

**References**

Andersson MF, Moller AM (2010) Assessment of carbon monoxide values in smokers: a comparison of carbon monoxide in expired air and carboxyhaemoglobin in arterial blood. European journal of anaesthesiology 27 (9):812-818. doi:10.1097/EJA.0b013e32833a55ea

Cander L, Forster RE (1959) Determination of pulmonary parenchymal tissue volume and pulmonary capillary blood flow in man. Journal of applied physiology 14 (4):541-551

Clemensen P, Christensen P, Norsk P, Gronlund J (1994) A modified photo- and magnetoacoustic multigas analyzer applied in gas exchange measurements. Journal of applied physiology 76 (6):2832-2839

Damgaard M, Norsk P (2005) Effects of ventilation on cardiac output determined by inert gas rebreathing. Clinical physiology and functional imaging 25 (3):142-147. doi:10.1111/j.1475-097X.2005.00602.x

Du Bois D, Du Bois EF (1916) Clinical calorimetry: tenth paper a formula to estimate the approximate surface area if height and weight be known. Archives of internal medicine 17 (6_2):863

Eastwood A, Hopkins WG, Bourdon PC, Withers RT, Gore CJ (2008) Stability of hemoglobin mass over 100 days in active men. Journal of applied physiology 104 (4):982-985. doi:10.1152/japplphysiol.00719.2007

Lang CC, Karlin P, Haythe J, Tsao L, Mancini DM (2007) Ease of noninvasive measurement of cardiac output coupled with peak VO2 determination at rest and during exercise in patients with heart failure. The American journal of cardiology 99 (3):404-405. doi:10.1016/j.amjcard.2006.08.047

Morgenthaler NG, Struck J, Jochberger S, Dünser MW (2008) Copeptin: clinical use of a new biomarker. Trends in Endocrinology & Metabolism 19 (2):43-49

Rossi GP, Barisa M, Belfiore A, Desideri G, Ferri C, Letizia C, Maccario M, Morganti A, Palumbo G, Patalano A, Roman E, Seccia TM, Pessina AC, Mantero F, Investigators Ps (2010) The aldosterone-renin ratio based on the plasma renin activity and the direct renin assay for diagnosing aldosterone-producing adenoma. Journal of hypertension 28 (9):1892-1899. doi:10.1097/HJH.0b013e32833d2192

Sackner MA, Feisal KA, Dubois AB (1964) Determination of Tissue Volume and Carbon Dioxide Dissociation Slope of the Lungs in Man. Journal of applied physiology 19:374-380
