# Supplementary material for: Interactions of the human cardiopulmonary, hormonal and body fluid systems in parabolic flight
Source: Eur J Appl Physiol. 2014 Mar 13;114(6):1281–95. doi: 10.1007/s00421-014-2856-3 (PMC4019836; doi:10.1007/s00421-014-2856-3)
Supplement: Supplementary file 3 — Online Resource 3.: Cardiovascular results of N = 18 as the mean ± SD of the parabolic flight tests are shown. (DOCX 16 kb) [file 421_2014_2856_MOESM3_ESM.docx]

| Parameter | Ground-Pre | Outbound | Gz | Block I | Block II | Block III | Block IV | Return | Ground-Post |
| --- | --- | --- | --- | --- | --- | --- | --- | --- | --- |
| HR | 100 ± 15 | 93 ± 18 | 0 | 92 ±15 | 81 ± 14 | 81 ± 15 | 78 ± 12 | 95 ± 11 | 91 ± 12 |
| (bpm) |  |  | 1.8 | 123 ± 21 | 113 ± 19 | 111 ± 17 | 110 ± 15 |  |  |
| FBP_syst_ | 127 ± 22 | 136 ± 21 | 0 | 144 ± 26 | 120 ± 30 | 121 ± 26 | 123 ± 22 | 124 ± 23 | 126 ± 14 |
| (mmHg) |  |  | 1.8 | 148 ± 21 | 132 ± 27 | 127 ± 24 | 131 ± 20 |  |  |
| FBP_diast_ | 72 ± 14 | 74 ± 14 | 0 | 70 ± 14 | 61 ± 17 | 60 ± 12 | 62 ± 12 | 76 ± 15 | 78 ± 10 |
| (mmHg) |  |  | 1.8 | 86 ± 14 | 85 ± 16 | 80 ± 14 | 83 ± 15 |  |  |
| FBP_mean_ | 90 ± 15 | 93 ± 15 | 0 | 94 ± 16 | 81 ± 17 | 80 ± 15 | 81 ± 14 | 91 ± 18 | 92 ± 10 |
| (mmHg) |  |  | 1.8 | 105 ± 14 | 101 ± 17 | 96 ± 15 | 96 ± 19 |  |  |
| SI_rb_ | 29 ± 8 | 33 ± 7 | 0 | 55 ± 11 | 58 ± 13 | 55 ± 8 | 53 ± 12 | 28 ± 6 | 26 ± 5 |
| $\left( \frac{ml}{m^{2}} \right)$ |  |  | 1.8 | 28 ± 8 | 24 ± 4 | ± 23 ± 4 | 24 ± 4 |  |  |
| CI_rb_ | 2.905 ± 0.678 | 3.052 ± 0.746 | 0 | 5.139 ± 1.326 | 4.742 ± 1.260 | 4.364 ± 1.038 | 4.150 ± 1.082 | 2.622 ± 0.542 | 2.564 ± 0.757 |
| $\left( \frac{L}{{min\times m}^{2}} \right)$ |  |  | 1.8 | 2.986 ± 0.743 | 2.653 ± 0.418 | 2.486 ± 0.411 | 2.677 ± 0.446 |  |  |
| SVR | 19 ± 5 | 18 ± 5 | 0 | 11 ± 3 | 11 ± 4 | 11 ± 3 | 12 ± 6 | 20 ± 6 | 23 ± 6 |
| $\left( \frac{mmHg}{L\times{min}} \right)$ |  |  | 1.8 | 19 ± 5 | 23 ± 5 | 23 ± 5 | 21 ± 5 |  |  |

**Online Resource #3**
