# Supplementary material for: Interactions of the human cardiopulmonary, hormonal and body fluid systems in parabolic flight
Source: Eur J Appl Physiol. 2014 Mar 13;114(6):1281–95. doi: 10.1007/s00421-014-2856-3 (PMC4019836; doi:10.1007/s00421-014-2856-3)
Supplement: Supplementary file 4 — Online Resource 4.: Pulmonary results of N = 18 as the mean ± SD of the parabolic flight tests are shown. (DOCX 15 kb) [file 421_2014_2856_MOESM4_ESM.docx]

| Parameter | Ground-Pre | Outbound | Gz | Block I | Block II | Block III | Block IV | Return | Ground-Post |
| --- | --- | --- | --- | --- | --- | --- | --- | --- | --- |
| SpO_2_ | 98 ± 0.6 | 95 ± 2 | 0 | 96 ± 2 | 95 ± 2 | 95 ± 2 | 95 ± 2 | 97 ± 2 | 97 ± 4 |
| (%) |  |  | 1.8 | 96 ± 1 | 95 ± 2 | 95 ± 2 | 95 ± 2 |  |  |
| Vt | 0.593 ± 0.181 | 0.688 ± 0.230 | 0 | 0.885 ± 0.458 | 0.737 ± 0.384 | 0.797 ± 0.642 | 0.729 ± 0.391 | 0.627 ± 0.230 | 0.542 ± 0.177 |
| (L) |  |  | 1.8 | 0.858 ± 0.357 | 0.870 ± 0.417 | 0.938 ± 0.562 | 0.783 ± 0.445 |  |  |
| VO_2_/kg | 3.9 ± 1.1 | 4.0 ± 1.2 | 0 | 9.6 ± 2.4 | 9.4 ± 2.8 | 8.8 ± 2.4 | 8.1 ± 2.6 | 3.2 ± 0.8 | 3.1 ± 1.0 |
| (ml) |  |  | 1.8 | 4.0 ± 1.2 | 3.3 ± 0.9 | 3.2 ± 1.0 | 3.3 ± 1.2 |  |  |

**Online Resource #4**
