# Supplementary material for: Interactions of the human cardiopulmonary, hormonal and body fluid systems in parabolic flight
Source: Eur J Appl Physiol. 2014 Mar 13;114(6):1281–95. doi: 10.1007/s00421-014-2856-3 (PMC4019836; doi:10.1007/s00421-014-2856-3)
Supplement: Supplementary file 5 — Online Resource 5.: Cardiovascular results as the mean ± SD of the hypobaric chamber tests are shown. N = 11 for both pulmonary and cardiovascular parameters. (DOCX 16 kb) [file 421_2014_2856_MOESM5_ESM.docx]

| Parameter | Ground-Pre | Outbound | Block I _1G_ | Block II _1G_ | Block III _1G_ | Block IV _1G_ | Return | Ground-Post |
| --- | --- | --- | --- | --- | --- | --- | --- | --- |
| Cardiovascular |  |  |  |  |  |  |  |  |
| HR  (bpm) | 81 ± 11 | 76 ± 9 | 76 ± 10 | 74 ± 9 | 76 ± 10 | 77 ± 8 | 79 ± 10 | 79 ± 10 |
| FBP_syst_  (mmHg) | 117 ± 16 | 123 ± 12 | 122 ± 14 | 124 ± 18 | 117 ± 14 | 118 ± 20 | 116 ± 18 | 124 ± 20 |
| FBP_diast_  (mmHg) | 68 ± 12 | 68 ± 10 | 71 ± 14 | 73 ± 18 | 70 ± 15 | 70 ± 19 | 68 ± 18 | 75 ± 18 |
| FBP_mean_  (mmHg) | 85 ± 12 | 86 ± 10 | 87 ± 14 | 89 ± 18 | 85 ± 15 | 85 ± 20 | 84 ± 18 | 90 ± 18 |
| SI_rb_  $\left( \frac{ml}{m^{2}} \right)$ | 31 ± 7 | 36 ± 10 | ±32 ± 8 | 35 ± 8 | ± 34 ± 8 | 34 ± 9 | 33 ± 8 | 30 ± 8 |
| CI_rb_  $\left( \frac{L}{{min\times m}^{2}} \right)$ | 2.509 ± 0.618 | 2.673 ± 0.714 | 2.423 ± 0.632 | 2.600 ± 0.679 | 2.561 ± 0.568 | 2.591 ± 0.695 | 2.538 ± 0.592 | 2.345 ± 0.561 |
| SVR  $\left( \frac{mmHg}{L\times{min}} \right)$ | 84.5 ± 12.2 | 86.0 ± 9.9 | 87.2 ± 14.2 | 89.4 ± 18.3 | 84.8 ± 14.7 | 84.7 ± 19.6 | 83.7 ± 18.0 | 90.3 ± 18.0 |
| Respiratory |  |  |  |  |  |  |  |  |
| SpO_2_  (%) | 98 ± 0.6 | 96 ± 2 | 96 ± 2 | 96 ± 2 | 96 ± 3 | 96 ± 2 | 96 ± 2 | 98 ± 1 |
| Vt  (L) | 0.694 ± 0.185 | 0.600 ± 0.243 | 0.560 ± 0.207 | 0.598 ± 0.226 | 0.562 ± 0.209 | 0.584 ± 0.214 | 0.535 ± 0.151 | 0.636 ± 0.171 |
| VO_2_/kg  (ml) | 3.0 ± 0.9 | 3.2 ± 0.9 | 3.0 ± 1.0 | 3.1 ± 1.1 | 3.1 ± 0.8 | 2.9 ± 1.0 | 3.1 ± 1.0 | 2.7 ± 0.9 |

**Online Resource #4**
