# Supplementary material for: Interactions of the human cardiopulmonary, hormonal and body fluid systems in parabolic flight
Source: Eur J Appl Physiol. 2014 Mar 13;114(6):1281–95. doi: 10.1007/s00421-014-2856-3 (PMC4019836; doi:10.1007/s00421-014-2856-3)
Supplement: Supplementary file 6 — Online Resource 6.: Plasma volume and biochemical responses in parabolic flight and the hypobaric chamber are given as the mean ± SD; # indicates parabolic flight values as the mean ± SD, which are presenting excluding the responses of the motion-sick subjects 0AP, 0AD and 0AT. The parabolic flight values of these subjects’ parameters are shown individually in Fig. 4. ‡, reninactive responses of subject 0AL in the hypobaric chamber were not included for mean and SD calculations because of an exceptional response. The 0AL-reninactive responses are shown individually in Fig. 4. (DOCX 16 kb) [file 421_2014_2856_MOESM6_ESM.docx]

| Parameter | Facility | Pre-Ground | Outbound | Post 16^th^ | Post 31^st^ | Post-Ground |
| --- | --- | --- | --- | --- | --- | --- |
| Plasma Volume | Airplane | 3375 ± 602 | 3414 ±592 | 3299 ±527 | 3330 ±571 | 3352 ±659 |
| (ml) | Chamber | 3126 ±732 | 3241 ±726 | 3067 ±662 | 3074 ±692 | 3206 ±704 |
| Albumin | Airplane | 46.3 ±2.9 | 46.0 ±3.1 | 47.3 ±2.7 | 46.9 ±4.2 | 47.9 ±2.8 |
| $\left( \frac{g}{L} \right)$ | Chamber | 45.7 ±3.0 | 44.4 ±2.2 | 47.0 ±9.4 | 46.5 ±2.4 | 45.1 ±2.5 |
| **^#^**Cortisol | Airplane | 17.4 ±3.7 | 12.9 ±4.0 | 13.2 ±4.5 | 12.3 ±6.2 | 11.0 ±4.8 |
| $\left( \frac{\mu g}{dl} \right)$ | Chamber | 14.0 ±6.7 | 11.9 ±4.1 | 10.6 ±3.1 | 10.4 ±4.0 | 10.2 ±3.3 |
| Aldosterone | Airplane | 126.5 ±51.7 | 146.2 ±58.0 | 160.2 ±74.1 | 157.9 ±70.5 | 165.5 ±64.9 |
| $\left( \frac{pg}{ml} \right)$ | Chamber | 157.0 ±78.9 | 154.0 ±78.9 | 168.5 ±80.8 | 155.2 ±78.0 | 137.1 ±77.9 |
| **^‡^**Renin_active_ | Airplane | 10.8 ±4.8 | 12.5 ±8.0 | 13.2 ±9.0 | 13.6 ±8.6 | 13.5 ±7.3 |
| $\left( \frac{pg}{ml} \right)$ | Chamber | 10.9 ±9.5 | 11.0 ±9.8 | 16.3 ±16.6 | 14.8 ±13.8 | 13.9 ±12.7 |
| Osmolality | Airplane | 312.1 ±15.1 | 310.8 ±10.8 | 310.7 ±14.0 | 312.1 ±16.2 | 308.8 ±13.3 |
| $\left( \frac{mosmol}{kg} \right)$ | Chamber | 300.5 ±4.9 | 301.2 ±5.3 | 300.5 ±4.0 | 299.8 ±4.2 | 298.7 ±4.4 |
| **^#^**CT-_pro_Vasopressin | Airplane | 9.4 ±11.7 | 6.3 ±7.2 | 5.3 ±5.1 | 8.5 ±10.0 | 4.9 ±3.8 |
| $\left( \frac{pmol}{L} \right)$ | Chamber | 4.2 ±3.4 | 3.4 ±1.6 | 3.2 ±1.9 | 4.1 ±2.8 | 3.7 ±2.2 |
| NT-_pro_BNP | Airplane | 65.9 ±52.5 | 66.9 ±50.5 | 72.9 ±57.1 | 73.6 ±51.6 | 66.8 ±45.9 |
| $\left( \frac{pg}{ml} \right)$ | Chamber | 60.6 ±43.9 | 58.7 ±39.3 | 57.5 ± 36.2 | 57.2 ±35.3 | 59.3 ±33.6 |

**Online Resource #5**
